# Treatment of Recurrent or Metastatic Uterine Adenosarcoma

**DOI:** 10.1155/2017/4680273

**Published:** 2017-12-28

**Authors:** Michael J. Nathenson, Anthony P. Conley, Heather Lin, Nicole Fleming, Vinod Ravi

**Affiliations:** ^1^Dana–Farber Cancer Institute, 450 Brookline Ave., Boston, MA 02215, USA; ^2^Department of Sarcoma Medical Oncology, The University of Texas MD Anderson Cancer Center, 1515 Holcombe Blvd Unit 450, Houston, TX 77030, USA; ^3^Department of Biostatistics, The University of Texas MD Anderson Cancer Center, 1515 Holcombe Blvd Unit 450, Houston, TX 77030, USA; ^4^Department of Gynecologic Oncology and Reproductive Medicine, The University of Texas MD Anderson Cancer Center, 1515 Holcombe Blvd Unit 450, Houston, TX 77030, USA

## Abstract

**Purpose:**

This study retrospectively evaluated overall survival (OS) by treatment of recurrent or metastatic uterine adenosarcoma including surgery, radiation, chemotherapy, and hormonal therapy and evaluated OS and progression-free survival (PFS) after 1st line systemic chemotherapy.

**Methods:**

78 patients with recurrent or metastatic adenosarcoma comprised the study population. The Kaplan-Meier method was used to estimate OS and PFS. The log-rank test was performed to test the difference in survival between groups.

**Results:**

Median OS from diagnosis of recurrent or metastatic disease was 1.8 yrs. OS was influenced by pathology on recurrence, *p*=0.035. Median OS differed by surgery for 1st recurrence 26.3 months versus 15.1 months. OS was not influenced by chemotherapy, *p*=0.58, palliative radiation, *p*=0.58, or hormonal therapy, *p*=0.15. The response rate (CR + PR) per RECIST 1.1 for chemotherapy was 31.2% for doxorubicin-based regimens and 14.3% for gemcitabine/docetaxel. OS since 1st line chemotherapy was not significantly different among chemotherapy regimens. However, the median PFS was superior for doxorubicin/ifosfamide (15.4 months) compared to gemcitabine/docetaxel (5.0 months), platinum-based regimens (5.7 mo), or other doxorubicin-based regimens (6.5 months).

**Conclusion:**

These results suggest that surgery is an important treatment modality for recurrent or metastatic uterine adenosarcoma, and the most effective chemotherapeutics are doxorubicin/ifosfamide and gemcitabine/docetaxel.

## 1. Introduction

Uterine adenosarcoma is an extremely rare subtype of uterine sarcoma, which represents only 5.5 to 9% of all uterine sarcomas [1, 2]. Uterine adenosarcoma was first described by Dr. Phillip B. Clement and Dr. Robert E. Scully in 1974 [3]. This tumor is composed of a malignant mesenchymal component and a benign epithelial component [4, 5]. This biphasic cellular differentiation is characteristic of adenosarcomas and required for the diagnosis of this tumor. Adenosarcomas can arise from the uterus but have also been noted to arise from the ovaries, vagina, cervix, and pelvis usually in the setting of prior endometriosis [6–9].

There is a specific FIGO uterine adenosarcoma staging system which divides between stage Ia, Ib, and Ic by the presence and extent of myometrial invasion [10]. The majority of adenosarcoma patients (70 to 80%) will present with stage I disease [11–13]. Despite this, many patients, even with stage I disease, will develop recurrent or metastatic disease. Survival varies with the presence and extent of myometrial invasion and sarcomatous overgrowth [12–15].

Unfortunately, given the rarity of adenosarcoma, there are limited data to guide treatment decisions in the recurrent or metastatic setting. There is no standard treatment for recurrent or metastatic disease, though surgery is often preformed [12]. A recent review suggests that uterine adenosarcomas can respond to doxorubicin/ifosfamide and gemcitabine/docetaxel chemotherapy [16]. Furthermore, the role of hormonal therapy in recurrent or metastatic disease is limited to case reports and case series.

The purpose of this study was to examine the role of surgery, radiation, and hormonal therapy in the recurrent or metastatic setting. Furthermore, this is the first study to report response rates, overall survival, and progression-free survival of adenosarcoma patients treated with chemotherapy for recurrent or metastatic disease.

## 2. Patients and Methods

With approval by the MD Anderson Cancer Center Institutional Review Board, a search was conducted of the Institutional Tumor Registry. Seventy-eight patients treated at the MD Anderson Cancer Center from August 1982 to December 2014 with recurrent or metastatic uterine or extrauterine adenosarcoma were identified. The diagnosis of uterine adenosarcoma was confirmed by MD Anderson sarcoma or gynecologic pathologists. Demographic, clinicopathologic, and treatment characteristics were abstracted from the patients' medical records. Then, a deidentified database was constructed of all adenosarcoma patients. The stage was assigned using the International Federation of Gynecology and Obstetrics 2009 uterine adenosarcoma staging system [10].

Patients' demographic and clinical characteristics were analyzed, with categorical variables summarized in frequency tables while continuous variables summarized using mean (±S.D.) and median (range). The product limit method of Kaplan and Meier was used to estimate overall survival (OS) and progression-free survival (PFS) [17]. OS was determined from the date of first recurrent or metastatic disease diagnosis to the date of death or date of last contact. OS since the start of chemotherapy for recurrent or metastatic disease was determined from the date of initiation of 1st line chemotherapy for recurrent or metastatic disease to the date of death or date of last contact. Progression-free survival was determined from the date of initiation of chemotherapy for recurrent or metastatic disease to the date of progression or death. Complete response (CR), partial response (PR), stable disease (SD), or progressive disease (PD) were determined by RECIST 1.1 [18]. The log-rank test was performed to test the difference in survival between groups [19]. Regression analyses of survival data utilized the Cox proportional hazards model [20].

## 3. Results

### 3.1. Patient Characteristics

Seventy-eight patients with recurrent or metastatic uterine or extrauterine adenosarcoma who received treatment at the MD Anderson Cancer Center were identified from a retrospective review of the Institutional Tumor Registry and were included in this study. The median follow-up time since recurrent or metastatic disease was 8.2 years. The demographic and clinical characteristics for all patients included in this study are summarized in Table 1. The median age at diagnosis was 55 years (range 27 to 79 years). The majority of patients were Caucasian (80.8%). The most common presenting symptom was abnormal uterine bleeding (44.9%), and the second most common presenting symptom was pelvic pain (16.7%). Thirty-nine patients had stage I primary tumor (50.1%). Thirty-six patients had stage II–IV primary tumor (46.2%). The primary tumor location was the uterine corpus in the majority of patients (74.4%). At last follow-up, 24 patients were alive, and 54 have died. Local recurrences occurred within the abdomen and pelvis in seventy-two patients, and sixteen patients developed distant metastasis. Patients who had local and distant recurrences commonly developed the local recurrence first. Sites of distant disease included the lung (14 pts), bone (4 pts), liver (3 pts), brain (1 pt), and subcutaneous tissue (4 pts).

### 3.2. Treatment Characteristics for Recurrent or Metastatic Disease

Treatment on recurrence varied greatly and was influenced by the location and extent of recurrence.  Table 2 describes the treatments that patients received on 1st and 2nd recurrence including surgery, palliative radiation, chemotherapy, and hormonal therapy. First-line chemotherapy for recurrent or metastatic disease was given to 59 patients, at 1st recurrence in 40 pts, at 2nd recurrence in 10 pts, at 3rd recurrence in 2 pts, and at 4th recurrence in 2 pts. Three additional patients had residual disease after primary treatment, and upon progression, they received 1st line chemotherapy (Table 2). One patient had 7 prior surgeries and hormonal therapy prior to receiving chemotherapy, and another patient received chemotherapy for 1st recurrence, though documentation regarding the specific chemotherapy regimen was lacking. The specific chemotherapy regimens utilized greatly differed as well.  Table 2 lists the specific chemotherapy regimens that patients received for 1st, 2nd, and 3rd line chemotherapy, as well as the number of patients that received surgery, radiation therapy, or hormonal therapy either before or after their chemotherapy as part of their treatment for recurrent or metastatic disease.

### 3.3. Pathology on Recurrence

**Figure 1:**
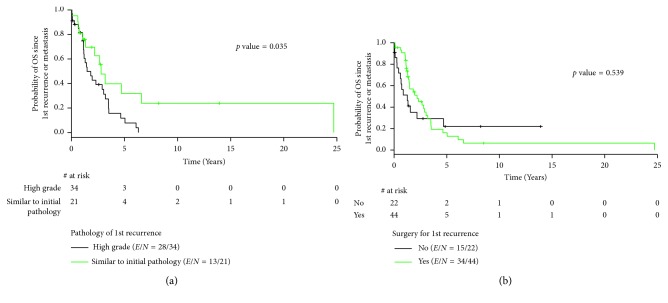
(a). Overall survival by pathology on recurrence. (b) Overall survival by surgery on recurrence.

On recurrence, 58 patients had a biopsy available for review: high-grade sarcoma in 36 patients and mixed epithelial and mesenchymal components in 22 patients. Median overall survival differed by the pathology of recurrence. Patients with high-grade sarcoma on recurrence had a median overall survival of 17.6 months versus 33.5 months in patients with mixed histology, *p*=0.035, HR = 0.47 (95% CI 0.23–0.96) (Figure 1(a) and Table 3).

### 3.4. Surgery for Recurrent or Metastatic Disease

Forty-five patients underwent surgery for recurrence or metastatic disease.

Forty-one patients underwent resection of recurrence within the abdomen and pelvis. Twenty-five patients underwent one resection, eight patients had two resections, four patients had three resections, three patients had four resections, and one patient had nine resections. Four patients underwent thoracotomies for resection of metastatic disease to the lung. Median overall survival was improved in those patients who underwent resection for recurrent or metastatic disease, for 1st recurrence 26.3 months versus 15.1 months, *p*=0.54 (Figure 1(b) and Table 3).

### 3.5. Chemotherapy for Recurrent or Metastatic Disease

#### 3.5.1. Response Rates

Of the fifty-nine patients that received 1st line chemotherapy for recurrent or metastatic disease, 9 received chemotherapy after surgical resection to obtain no evidence of disease, so response rate per RECIST 1.1 could not be calculated. Four patients received only 1 cycle of chemotherapy and were excluded. Imaging was not accessible for review in another twenty-five patients. Twenty-one patients with chemotherapy for 1st line recurrent or metastatic disease were evaluable for response per RECIST 1.1 (Table 4). The complete response (CR) + partial response (PR) rate for doxorubicin-based 1st line chemotherapy was 40% versus 25% for gemcitabine/docetaxel. Another 14 patients received 2nd or 3rd line chemotherapy and were evaluable for response per RECIST 1.1. The CR + PR rate for doxorubicin-based 1st, 2nd, and 3rd line chemotherapy was 31.2% versus 14.3% for gemcitabine/docetaxel (Table 3). The majority of the responses with doxorubicin-based regimens were seen in patients receiving the combination of doxorubicin and ifosfamide (4/5 pts) or doxorubicin and dacarbazine (1/5 pts). The rate of CR + PR + stable disease (SD) was 87.5% for doxorubicin-based regimens and only 42.9% for gemcitabine/docetaxel. Only five patients with a platinum-based regimen were evaluable for RECIST 1.1, 80% had SD, and the one patient with a partial response, also, received ifosfamide.

### 3.6. Survival Analysis

#### 3.6.1. Overall Survival and Progression-Free Survival

**Figure 2:**
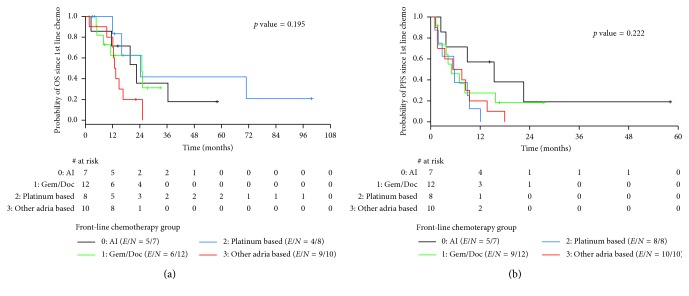
(a) and (b) Overall survival and progression-free survival for 1st line chemotherapy for recurrent or metastatic disease.

The median overall survival after 1st recurrence was 1.8 yrs. Table 3 shows the median overall survival and progression-free survival from the beginning of 1st line chemotherapy for patients receiving 1st line chemotherapy for recurrent or metastatic disease. Thirty-seven patients were evaluable for time-to-event outcomes. Nine patients received only one cycle of chemotherapy and were excluded from these analyses. The median overall survival from the beginning of 1st line chemotherapy was 16.6 months. Median overall survival differed by the chemotherapy regimen (Figure 2(a)). Median overall survival was worse for all doxorubicin-based regimens, 14.9 months, when compared to gemcitabine/docetaxel, 24.9 months, or platinum-based chemotherapy, 24.3 months. The median overall survival for doxorubicin/ifosfamide (AI) was 22.5 months, not statistically different from gemcitabine/docetaxel (*p*=0.99) or platinum-based regimens (0.49). The median overall survival for doxorubicin-based regimens, other than AI, was 13.1 months. Additionally, patients who received AI had improved PFS (15.4 months) compared to other doxorubicin-based regimens (6.5 months), gemcitabine/docetaxel (5.0 mo), and platinum-based regimens (5.7 months) (Figure 2(b)). This difference trended toward statistical significance when comparing AI to other doxorubicin-based regimens *p*=0.083 or to platinum-based regimens *p*=0.06.

Patients were analyzed for other factors which may have influenced the effectiveness of their chemotherapeutic regimens, such as chemotherapy dosing, # of chemotherapy cycles, presence of sarcomatous overgrowth (SO), point in their disease course when they received chemotherapy, and time to recurrence prior to initiation of chemotherapy, a potential indicator of the aggressiveness of their disease. Patients did not significantly differ in terms of chemotherapy dosing, # of chemotherapy cycles, presence of SO, or point in their disease course when they received chemotherapy. The median time to recurrence prior to initiation of 1st line chemotherapy was 10.1 months for all doxorubicin-based regimens, 12.9 months for AI, 9.6 months for other doxorubicin-based regimens, 8.1 months for platinum-based regimens, and 21.7 months for gemcitabine/docetaxel, indicating a possible physician bias for treating patients with doxorubicin-based regimens in patients with quicker relapses, so more aggressive disease.

### 3.7. Hormonal Therapy for Recurrent or Metastatic Disease

Twenty-eight patients received hormonal therapy at some point during their treatment course. Seven patients received more than 1 line of hormonal therapy. Initial hormonal therapies included GnRH agonists (leuprolide, 9 pts), progesterones (megestrol acetate, 7 pts), SERMs (tamoxifen 3 pts and raloxifene 1 pt), and aromatase inhibitors (anastrozole 3 pts and letrozole 1 pt). The medial overall survival for patients with recurrent disease who received hormonal therapy was 34.7 months compared to 17.6 months, a trend toward improved outcomes that was not statistically significant, *p*=0.15.

Out of these twenty-eight patients, there were four that derived several years of benefit from hormonal therapy. Two patients treated with leuprolide had disease control for >2 years. Their tumors were not stained for the estrogen receptor (ER) or progesterone receptor (PR). A third patient was placed on leuprolide after surgical resection with the development of progression after two years. This patient was then placed on megestrol acetate without response and then on anastrozole with a complete response lasting for eight years. This patient developed a 2nd malignancy. She was taken off anastrozole at the time of surgery for her cholangiocarcinoma. Shortly after completing adjuvant chemotherapy for her cholangiocarcinoma, she developed recurrence of her adenosarcoma, biopsy proven. She was placed back on anastrozole with response lasting for another five years. More recently, she developed progression and is now on systemic chemotherapy with trabectedin. This patient's tumor stained for ER 80% and PR 40%. The mesenchymal portion of her initial tumor was described as endometrial stromal sarcoma, though she did have sarcomatous overgrowth noted as well. A fourth elderly patient with locally advanced unresectable disease was treated with leuprolide and carboplatin for seven cycles leading to a partial response. Her leuprolide was continued after chemotherapy resulting in a complete response after five years with resolution of her pelvic mass and remaining subcentimeter pelvic lymphadenopathy. She continued leuprolide for 14 years, at which point her leuprolide was stopped. She then developed radiographic recurrence within the abdomen and was restarted on leuprolide with stable disease until her death at age 95. The ER/PR status and SO status of her tumor are unknown.

## 4. Discussion

In this study, we report the largest single-institution experience with recurrent or metastatic uterine and extrauterine adenosarcoma. There is no standard treatment for patients with local recurrence or metastatic uterine adenosarcoma. Treatment options include surgery, radiation, chemotherapy, and hormonal therapy. This study is the first to show that the pathology on recurrence influences outcomes. Specifically, those patients that present with a recurrence that is purely high-grade sarcoma have significantly worse outcomes than those patients who present with a recurrent tumor with mixed epithelial and mesenchymal components. This may represent selective clonal evolution of these tumors such that patients with a recurrence of pure high-grade sarcoma have a more clinically aggressive course.

The majority of uterine adenosarcomas recur locally, suggesting that resection of a local recurrence may improve overall survival and time to next recurrence. Previous reports have indicated a benefit for secondary cytoreduction of recurrent adenosarcoma in terms of overall survival and time to next recurrence [12, 21]. Our study shows an improvement in overall survival for those patients that underwent surgery for 1st recurrence, though this was not statistically significant. There is a selection bias in this result, in that patients able to undergo surgery likely had better functional status at the time of surgery, less medical comorbidities, and recurrence amenable to surgical resection. However, given the improvement in overall survival, it may be worth considering surgical resection of a recurrence, for those patients amenable to surgery.

This is the first retrospective report to examine the use of systemic chemotherapy in a large population of recurrent uterine adenosarcoma. Case reports and case series have described responses in adenosarcoma with the use of doxorubicin-based regimens [21–26], gemcitabine/docetaxel [21, 27], trabectedin [28], or platinum-based regimens [21]. This report shows that active agents in uterine adenosarcoma are doxorubicin/ifosfamide (AI), doxorubicin/dacarbazine (ADIC), and gemcitabine/docetaxel. The most effective agents for adenosarcoma in terms of response per RECIST 1.1 were the combination of doxorubicin and ifosfamide. All three are reasonable chemotherapeutic choices for recurrent or metastatic uterine adenosarcoma. The dosing of chemotherapy in this retrospective study varied greatly; however, the patients that received gemcitabine/docetaxel received 675 to 900 mg/m^2^ of gemcitabine and 75 to 100 mg/m^2^ of docetaxel; the patients that received doxorubicin-based regimens received 60 to 75 mg/m^2^ of doxorubicin, 7.5 to 10 gm/m^2^ of ifosfamide, and 750 to 1000 mg/m^2^ of dacarbazine. This is standard sarcoma chemotherapy dosing.

The OS after 1st line chemotherapy for recurrent or metastatic disease was not statistically different between AI, gem/doc, or platinum-based regimens. However, OS may be affected by subsequent therapies such as surgery, hormonal therapy, or further chemotherapy. Additionally, there was a trend toward worse survival for patients that received other doxorubicin-based regimens excluding AI, suggesting that ifosfamide is required to obtain the higher response rate and clinical benefit. However, whether this effective is limited to ifosfamide alone or a result of synergy between doxorubicin/ifosfamide, as noted in soft tissue sarcomas, is undetermined [29].

PFS may be a better measure of benefit from systemic chemotherapy. Patients who received AI had the longest PFS (15.4 months), with a trend toward statistical significance when compared to patients who received doxorubicin alone or in combination with a 2nd agent or platinum-based regimens. If the goal of therapy is to produce reduction in tumor size prior to surgical resection of recurrent disease, AI chemotherapy may have advantages over gemcitabine/docetaxel. If patients are older with poor function status and multiple medical comorbidities, they are not a doxorubicin/ifosfamide candidate; then gemcitabine/docetaxel may be the preferred regimen. Cisplatin- and carboplatin-based regimens appear to be the least effective in uterine adenosarcomas and should not be recommended for treatment of recurrent or metastatic disease. Further study is required to evaluate the role of trabectedin in the treatment of uterine adenosarcomas.

It should be noted that there was likely a physician bias in choosing treatment with doxorubicin-based regimens over gemcitabine/docetaxel. There was a shorter median time to recurrence prior to the start of 1st line chemotherapy with AI (12.9 months) or other doxorubicin-based regimens (9.6 months) compared to gemcitabine/docetaxel (21.7 months), indicating that physicians were more likely to treat with doxorubicin-based regimens than with gemcitabine/docetaxel for patients that had quicker relapses, or more aggressive disease. This suggests a benefit of doxorubicin/ifosfamide over gemcitabine/docetaxel in the treatment of uterine adenosarcomas, despite similar median OS, as patients with more aggressive disease would be expected to have worse survival.

Evidence for the use of hormonal therapy in uterine adenosarcoma is even more limited than evidence for the use of chemotherapy. Case reports or case series have noted responses to hormonal therapy lasting 10 months to 7 years [12, 30–33]. Agents used include GnRH agonists, progesterones, selective estrogen receptor modulators, or aromatase inhibitors. Responses have been occasionally correlated with the presence of estrogen receptor (ER) and progesterone receptor (PR) staining. Loss of ER and PR expressions has been associated with sarcomatous overgrowth [34]. Furthermore, loss of response to hormonal therapy was associated with reduced ER/PR expression in one case report [33]. This suggests ER and PR as possible predictors of response to hormonal therapy in uterine adenosarcomas, though this has not been studied in a systematic manner. In this study, the majority of patients (86%) did not receive benefit from hormonal therapy, though there were 4 patients that derived benefit in terms of stable disease and improved survival from leuprolide or anastrozole for 2 to 15 years, suggesting that select patients may have a benefit from hormonally targeted therapy. Unfortunately, we are currently unable to determine which patients will receive such a large benefit from hormonal therapy.

This study is limited by its retrospective nature and small sample size, though this is the largest single-institution recurrent or metastatic uterine or extrauterine adenosarcoma series to date. Overall, uterine adenosarcoma is a rare disease with limited evidence-based data to determine treatment recommendations. Treatment of recurrence or metastatic disease can consist of surgery, chemotherapy, preferable with doxorubicin/ifosfamide or gemcitabine/docetaxel, or hormonal therapy in select patients.

## Figures and Tables

**Table 1:** Patient characteristics.

Variable	Patients with recurrence, no. of patients (%)
Age, median (range)	55 years (27 to 79 years)
Race	
Caucasian	63 (80.8%)
African American	10 (12.8%)
Hispanic	5 (6.4%)
ECOG performance status	
0	17 (21.8%)
1	30 (38.5%)
Unknown	31 (39.7%)
Presenting symptoms	
Abnormal uterine bleeding	35 (44.9%)
Pelvic pain	13 (16.7%)
Incidental finding	4 (5.1%)
Other	6 (7.7%)
Unknown	20 (25.6%)
Size, median (range)	7.1 cm (0.4 to 25 cm)
≤5 cm	14 (17.9%)
>5 cm	36 (46.2%)
Unknown	28 (35.9%)
Myometrial invasion	
No myometrial invasion	12 (15.4%)
≤½ of myometrium	26 (33.3%)
>½ of myometrium	8 (10.3%)
Unknown	32 (41.0%)
Sarcomatous overgrowth	
Absent	15 (19.2%)
Present	58 (74.4%)
Unknown	5 (6.4%)
Lymph node involvement	
None	34 (43.6%)
Present	1 (1.3%)
No lymph node sampling	39 (50.0%)
Unknown	4 (5.1%)
Uterine adenosarcoma FIGO stage at diagnosis	
Ia	7 (9.0%)
Ib	19 (24.4%)
Ic	4 (5.1%)
I (nos.)	9 (11.6%)
IIa	7 (9.0%)
IIb	12 (15.4%)
IIIa	7 (9.0%)
IIIb	4 (5.1%)
IIIc	1 (1.3%)
IVa	4 (5.1%)
IVb	1 (1.3%)
Unknown	3 (3.8%)
Tumor location	
Uterine corpus	58 (74.4%)
Ovary	5 (6.4%)
Cervix	2 (2.6%)
Pelvic primary	11 (14.1%)
Vagina	1 (1.3%)
Unknown	1 (1.3%)
Initial treatment strategy	
Surgery alone	45 (57.7%)
Surgery + radiation (adjuvant or neoadjuvant)	22 (28.2%)
Surgery + chemotherapy (adjuvant or neoadjuvant)	6 (7.7%)
Surgery + radiation + chemotherapy	4 (5.1%)
Surgery + hormonal therapy	1 (1.3%)
Bilateral salpingo-oophorectomy	
Yes	55 (70.5%)
No	13 (16.7%)
History of prior BSO	9 (11.5%)
Unknown	1 (1.3%)
Lymphadenectomy	
Yes	31 (39.7%)
No	43 (55.1%)
Unknown	4 (5.1%)
Vital status	
Alive	24 (30.8%)
Dead	54 (69.2%)
Recurrence	
Any recurrence	78
Local (abdomen/pelvis)	61 (78.2%)
Local and distant	11 (14.1%)
Distant	5 (6.4%)
Unknown	1 (1.3%)
Median follow-up	8.2 years

This table described patient demographics (age and race), presenting symptoms, performance status, tumor pathologic characteristics (size, myometrial invasion, sarcomatous overgrowth, and lymph node involvement), tumor stage, tumor location, initial treatments (surgery, radiation, chemotherapy, BSO, and lymphadenectomy), vital status (alive versus dead), and recurrence location for patients with recurrence.

**Table 2:** Treatment characteristics on recurrence with 1st, 2nd, and 3rd line chemotherapy for recurrent or metastatic disease.

Treatment on 1st recurrence	
Surgery alone	13 (16.7%)
Surgery + radiation	8 (10.3%)
Radiation alone	1 (1.3%)
Surgery + chemotherapy	19 (24.3%)
Chemotherapy alone	18 (23.1%)
Surgery + chemotherapy + radiation	5 (6.4%)
Chemotherapy + radiation	1 (1.3%)
Hormonal therapy alone	3 (3.8%)
None	1 (1.3%)
Unknown	9 (11.6%)
Treatment on 2nd recurrence	
Surgery alone	11 (14.1%)
Surgery + radiation	2 (2.6%)
Surgery + chemotherapy	6 (7.7%)
Chemotherapy alone	11 (14.1%)
Chemotherapy + radiation	2 (2.6%)
Unknown	17 (21.8%)
Did not obtain NED after 1st recurrence	29 (37.2%)
1st line chemotherapy additional treatments	
Chemotherapy alone	30 (50.9%)
Chemotherapy + surgery	20 (33.9%)
Chemotherapy + radiation	2 (3.4%)
Chemotherapy + radiation + surgery	4 (6.8%)
Chemotherapy + hormonal therapy	1 (1.7%)
Chemotherapy + surgery + hormonal therapy	2 (3.4%)
Chemotherapy given for residual disease after initial treatment	3 (5.1%)
Chemotherapy given for 1st recurrence	40 (67.8%)
Chemotherapy given for 2nd recurrence	10 (16.9%)
Chemotherapy given for 3rd recurrence	2 (3.4%)
Chemotherapy given for 4th recurrence	2 (3.4%)
Chemotherapy given for >4th recurrence	1 (1.7%)
Recurrence chemotherapy given for unknown	1 (1.7%)
1st line chemotherapy regimens	
Doxorubicin based	28 (47.5%)
Doxorubicin alone^+^	4
AI (doxorubicin/ifosfamide)	9
VAI (vincristine/doxorubicin/ifosfamide)	1
ADIC (doxorubicin/dacarbazine)	3
MAID (doxorubicin/ifosfamide/dacarbazine)	1
Other doxorubicin based^∗^	10
Gemcitabine/docetaxel	14 (23.7%)
Platinum based^∗∗^	12 (20.3%)
Other (ifosfamide/paclitaxel (2), paclitaxel, gemcitabine)	4 (6.8%)
Unknown	1 (1.7%)
2nd line chemotherapy regimens	
Doxorubicin based	6 (25.0%)
Doxorubicin alone^+^	3
AI (doxorubicin/ifosfamide)	3
Gemcitabine/docetaxel	9 (37.5%)
Platinum based (cisplatin/ifosfamide in one patient)^∗∗∗^	4 (16.7%)
Other (ifosfamide, paclitaxel, paclitaxel/bevacizumab, irinotecan/dacarbazine, trabectedin)	5 (20.8%)
3rd line chemotherapy regimens	
Doxorubicin based	4 (36.4%)
Doxorubicin alone	2
AI (doxorubicin/ifosfamide)	1
ADIC (doxorubicin/dacarbazine)	1
Platinum based^∗∗∗∗^	2 (18.2%)
Other (ifosfamide (2), paclitaxel, I/E^++^, TMZ^+++^)	5 (45.5%)

Chemotherapy dosing: gemcitabine 675 to 900 mg/m^2^, docetaxel 75 to 100 mg/m^2^, vincristine 2 mg, doxorubicin 60 to 75 mg/m^2^, ifosfamide 7.5 to 10 gm/m^2^, and dacarbazine 750 to 1000 mg/m^2^; ^∗^doxorubicin/cyclophosphamide/cisplatin (1), low-dose doxorubicin/ifosfamide (50 mg/m^2^, 2.4 gm/m^2^) (1), doxorubicin/cisplatin (1), vincristine/doxorubicin/cyclophosphamide (1), doxorubicin/cisplatin/paclitaxel (1), doxorubicin/dacarbazine/cyclophosphamide (3), doxorubicin/carboplatin (1), and vincristine/doxorubicin/cyclophosphamide/dacarbazine (1); ^∗∗^carboplatin/docetaxel (2), bleomycin/etoposide/cisplatin (2), carboplatin/paclitaxel (4), cisplatin with weekly radiation (2), carboplatin/paclitaxel/bevacizumab (1), and carboplatin/cyclophosphamide (1); ^∗∗∗^carboplatin/docetaxel (1), carboplatin/paclitaxel (1), cisplatin/ifosfamide (1), and carboplatin/gemcitabine (1); ^∗∗∗∗^carboplatin/gemcitabine (1) and cisplatin/gemcitabine (1); ^+^one patient had liposomal doxorubicin; ^++^ifosfamide/etoposide; ^+++^temozolomide.

**Table 3:** Treatment outcomes on recurrence with chemotherapy for recurrent or metastatic disease.

	Median survival	*p* value	HR	95% CI
OS by pathology on recurrence				
High-grade sarcomatous component only	17.6 mo			
Similar to initial pathology	33.5 mo	0.035	0.47	0.23–0.96
1st line chemotherapy				
OS				
All patients	16.6 mo	—		
Doxorubicin-based regimen	14.9 mo	—		
Doxorubicin/ifosfamide	22.5 mo	ref		
Other doxorubicin-based regimens	13.1 mo	0.18	2.18	0.69–6.88
Gemcitabine/docetaxel	24.9 mo	0.99	0.99	0.30–3.38
Platinum-based regimen	24.3 mo	0.49	0.60	0.14–2.53
PFS				
All patients	7.0 mo	—		
Doxorubicin-based regimen	8.5 mo	—		
Doxorubicin/ifosfamide	15.4 mo	ref		
Other doxorubicin-based regimens	6.5 mo	0.083	2.65	0.88–7.98
Gemcitabine/docetaxel	5.0 mo	0.27	1.87	0.62–5.63
Platinum-based regimen	5.7 mo	0.06	3.08	0.95–9.93
1st line chemotherapy additional treatments				
OS				
Surgery	21.6 mo			
No surgery	11.9 mo	0.12	1.36	0.73–2.53
PFS				
Surgery	12.0 mo			
No surgery	3.6 mo	0.02	2.04	1.10–3.77
OS by hormonal therapy for patients with recurrence				
Received hormonal therapy	34.7 mo			
No hormonal therapy	17.6 mo	0.15	1.58	0.85–2.95
OS therapy for 1st recurrence	21.8 mo			
Surgery	26.3 mo			
No surgery	15.1 mo	0.54	1.21	0.66–2.24
Radiation	16.5 mo			
No radiation	23.4 mo	0.58	0.83	0.42–1.63
Chemotherapy	18.6 mo			
No chemotherapy	27.6 mo	0.58	0.85	0.47–1.53
Chemotherapy + surgery	23.4 mo			
No chemotherapy + surgery	16.5 mo	0.97	1.01	0.55–1.85

OS, overall survival; PFS, progression-free survival; TTP, time to progression.

**Table 4:** Response rates with chemotherapy for recurrent or metastatic disease.

	1st line chemotherapy	All lines of chemotherapy (1, 2, 3)
Doxorubicin-based regimens		
CR	2 (20%)	3 (18.7%) (AI (2), ADIC)
PR	2 (20%)	2 (12.5%) (AI, VAI)
(CR + PR)	4 (40%)	5 (31.2%)
SD	5 (50%)	9 (56.3%) (AI (4), ADIC, Dox (3), CyA)
(CR + PR + SD)	9 (90%)	14 (87.5%)
PD	1 (10%)	2 (12.5%) (AI, Dox)
Total patients	10	16
Gemcitabine/docetaxel		
CR	1 (12.5%)	1 (7.1%)
PR	1 (12.5%)	1 (7.1%)
(CR + PR)	2 (25%)	2 (14.3%)
SD	3 (37.5%)	6 (42.9%)
(CR + PR + SD)	5 (62.5%)	8 (57.1%)
PD	3 (37.5%)	6 (42.9%)
Total patients	8	14
Platinum-based regimens		
CR	0	0
PR	0	1 (20%) (Cis/Ifos)
(CR + PR)	0	1 (20%)
SD	3 (100%)	4 (80%) (Cis/Gem, Cis + XRT, carbo/taxol (2))
(CR + PR + SD)	3 (100%)	5 (100%)
PD	0	0
Total patients	3	5

CR, complete response; PR, partial response; SD, stable disease; PD, progressive disease; AI, doxorubicin/ifosfamide; ADIC, doxorubicin/dacarbazine; VAI, vincristine/doxorubicin/ifosfamide; Dox, doxorubicin; CyA, doxorubicin/cyclophosphamide; Cis, cisplatin; Ifos, ifosfamide; Gem, gemcitabine; carbo, carboplatin; XRT, radiation; taxol, paclitaxel.
